# Update on gene fusions and the emerging clinicopathological landscape of peritoneal and pleural mesotheliomas and other neoplasms

**DOI:** 10.1016/j.esmoop.2024.103644

**Published:** 2024-07-25

**Authors:** N. Benzerdjeb, P. Dartigues, V. Kepenekian, F. Damiola, R. Sequeiros, F. Galateau-Salle, H. Begueret, E. Mery, D. Damotte, V. Verriele, J. Fontaine, S. Isaac, S. Valmary-Degano, L. Villeneuve, O. Glehen, A. Scherpereel, F. Forest, A. De la Fourchardiere, S. Paindavoine, A. Hourlier, D. Pissaloux, F. Tirode, S. Lantuejoul, RENAPE and the NETMESO/MESOPATH Networks

**Affiliations:** 1 Department of Pathology, Institut de Pathologie Multisite, Lyon-Sud University Hospital, Hospices Civils de Lyon, Pierre-Bénite; 2 CICLY - EA3738, Université Claude Bernard Lyon 1, Lyon; 3 Department of Pathology, Gustave Roussy Institute, Villejuif; 4 Department of Digestive Surgery, CNR RENAPE, Lyon-Sud University Hospital, Lyon; 5 The Unit of Molecular Pathology, INSERM 1052, CNRS 5286 of Cancer Research Center of Lyon, and Team Genetics, Epigenetics and Biology of Sarcomas, Université Claude Bernard Lyon 1, Lyon; 6 Department of Biopathology, CNR MESOPATH NETMESO, CLCC UNICANCER Leon Berard, Lyon; 7 Department of Pathology, Bordeaux University Hospital, Bordeaux; 8 Department of Pathology, Claudius Regaud Institute, IUTC Oncopôle, Toulouse; 9 Department of Pathology, Centre - Paris University Hospital, Cochin Hospital, Assistance Publique - Hôpitaux de Paris, Paris; 10 Centre de Recherche des Cordeliers, University Sorbonne, INSERM, University Paris Cité, Team Inflammation, Complement and Cancer, Paris; 11 Institut de Cancérologie de l’Ouest, Angers; 12 University Grenoble Alpes, Inserm U1209, IAB, Department of Pathology, University Hospital, Grenoble; 13 Department of Epidemiology and Clinical Research, Pôle de Santé Publique, Hospices Civils de Lyon, Lyon; 14 University of Lille, Thoracic Oncology Department, CNR Mesoclin NETMESO, CHU Lille CNRS, INSERM, Institut Pasteur de Lille, UMR9020-UMR-S 1277-Canther, Lille; 15 Department of Pathology, University Hospital of Saint Etienne, Saint Etienne; 16 University Grenoble Alpes, Grenoble, France

**Keywords:** *ALK*, *EWSR1*, *MAP3K8*, *SUFU*, gene fusion, mesothelioma

## Abstract

**Background:**

Mesothelioma is a rare and aggressive malignant neoplasm arising from mesothelial cells, which occasionally manifests recurrent fusions. *EWSR1*/*FUS*-*CREB*, *YY1*, *MAP3K8*, *NR4A3*, and *ALK*-rearranged proliferations have been reported in limited series with no clear histological or clinical correlations, limiting clinicians’ ability to assess prognosis and integrate these new entities into therapeutic decisions. The aim of this study was to better characterize these rearranged proliferations histologically, molecularly, and clinically.

**Methods:**

Clinical, pathological, and comprehensive transcriptome and mutation data were collected for each case.

**Results:**

A total of 41 tumors were included, encompassing 7 *ALK*, 10 *MAP3K8*, 4 *NR4A3*, 8 *ESWR1*/*FUS*::*ATF1*, 8 *EWSR1*::*YY1*, and 4 *SUFU*-fused cases. We found a female predominance, except for cases harboring *NR4A3* and *SUFU*; and most patients were around 60 years of age, but those harboring *ALK* or *EWSR1/FUS::ATF1* gene fusions were younger. Each group exhibited distinct histological, immunohistochemical, molecular features, and oncological courses. Specifically, *MAP3K8* and *ALK* presented PAX8+ papillary proliferations, *ESWR1*/*FUS*::*ATF1* and *EWSR1*::*YY1* displayed angiomatoid fibrous histiocytoma-like patterns, while *SUFU* showcased ‘tissue culture’-like spindle cell proliferation. Poor prognosis factors were the pleural site, male sex, Ki67 ≥10%, and *ESWR1*/*FUS*::*ATF1* or *SUFU* gene fusions.

**Conclusions:**

This study significantly broadens the spectrum of mesothelial tumors associated with fusions, offering insight into novel epithelioid (mesothelial) proliferations with distinctive histological appearances, molecular profiles, and prognoses to guide adapted treatments for patients.

## Highlights

• Investigation histologically, molecularly, and clinically of 41 peritoneal and pleural rearranged mesotheliomas and neoplasms.
• Each group exhibited distinct histological, immunohistochemical, molecular features, and overall survival.
• A female predominance was found, except for cases of *NR4A3* and *SUFU* fusion.
• Most patients were aged 60 years, but those harboring *ALK* or *EWSR1/FUS::ATF1* gene fusions were younger.
• A longer overall survival was found for female sex, peritoneum or tunica vaginalis testis site, *ALK*, or *NR4A3* fusions, and Ki67 <10%.

## Graphical abstract

**Figure:**
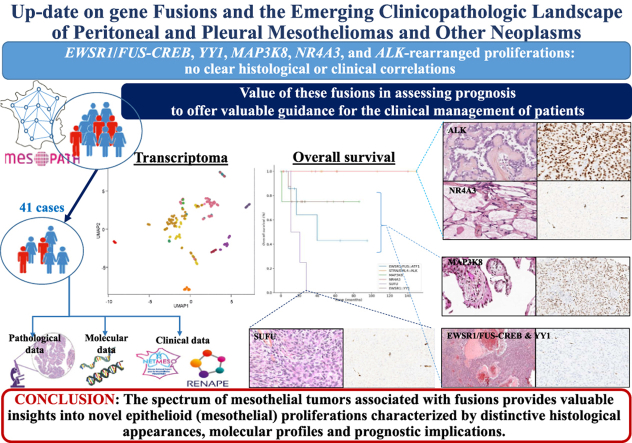

## Introduction

Mesotheliomas are uncommon malignant tumors originating from mesothelial cells that line various serous cavities, including pleura, pericardium, peritoneum, and tunica vaginalis testis.^1^^,^^2^ These tumors are categorized into three primary histological types: epithelioid (EM), sarcomatoid (SM), and biphasic types, the epithelioid type representing in the pleura nearly 75% of the cases, the biphasic type 15% and the sarcomatoid type up to 10%.^3^ Over the last decade, significant strides have been made in understanding mesothelioma pathogenesis. This progress has been driven by the integration of cutting-edge molecular and genomic techniques into research endeavors. As a result, numerous studies have embarked on the journey to unravel the genetic factors and oncogenic pathways at the core of mesothelioma, all in pursuit of discovering fresh avenues for therapeutic intervention.^4^, ^5^, ^6^ Recently, small series of mesothelioma, mostly from the peritoneum, were reported to be driven by recurrent gene fusions, such as *EWSR1/FUS-CREB*, *YY1*, *MAP3K8*, *NR4A3* and *ALK* fusions,^7^, ^8^, ^9^, ^10^, ^11^, ^12^, ^13^, ^14^, ^15^, ^16^, ^17^, ^18^, ^19^ but most often with no specific clinical or histological features. To better characterize these rearranged proliferations, our group carried out a clinicopathological and further molecular analysis of 41 cases previously identified as harboring a fusion in comparison with 160 control cases without fusion, all collected from the RENAPE and NETMESO/MESOPATH networks.

## Patients and methods

### Population

The cohort consisted of 41 patients treated for rearranged mesotheliomas/neoplasms; the control group included 160 patients treated for neoplasms without rearrangement, the slides for whom were reviewed by the expert pathologists of the Rare Peritoneal Tumors network (RENAPE), and/or the Pleural Mesothelioma and Other Rare Pleural Tumors network (NETMESO/MESOPATH), according to the standardized procedure of certification. One sample was used for each patient. Tumors with clinical annotations were retrieved from the French MESOBANK and its clinicopathological database. Clinical data recorded were age at the time of diagnosis, sex, size, site and number of the lesions, treatment by surgery and/or chemotherapy, duration of follow-up, and survival. The histopathological criteria analyzed were architecture, cellular size and shape, nuclear grade and shape, necrosis, infiltration of the underlying structures, and tumor microenvironment.

### Governance

Tissue samples from the French MESOBANK were collected in agreement with all applicable laws, rules, and requests of French and European authorities. These samples were prepared by Centre de Ressources Biologiques of Leon Berard Cancer Center [BB-0033-00050, Lyon France, whose activity is declared to the Ministry of Research (DC-2008-99 and AC-2019-3426)]. This study was approved by the review board of the MESOBANK (number 2021-11-17-MesoFusion) and is compliant with General Data Protection Regulation requirements and the French national data protection commission (Commission nationale de l’informatique et des libertés, CNIL; number R201-004-241 on 31 March 2022).

### Histological examination

All slides [including hematoxylin, phloxine, and saffron stain, and immunohistochemistry (IHC) staining] were reviewed by two independent pathologists (NB and SL) and classified according to the 2021 WHO classification.^20^

### Immunohistochemistry

A three-step IHC was carried out on 4 μm-thick formalin-fixed paraffin-embedded (FFPE) tissue sections on a BenchMark Ventana autostainer in the biopathology department of the Leon Berard Cancer Center. The antibodies used were raised against pan-cytokeratins, CK5/6, calretinin, WT1, BAP1, CEA, BerEP4, ERα, and PAX8. The proliferation index was assessed by Ki67 nuclear staining (MiB1 clone). The source and dilutions of the antibodies are listed in Supplementary Table S1, available at https://doi.org/10.1016/j.esmoop.2024.103644. The tumor immunophenotype was considered as classical of mesothelial differentiation when calretinin, CK5/6, and/or WT1 were found positive, and BerEp4/CEA negative. Conversely, a doubtful profile of mesothelial differentiation was considered when calretinin, CK5/6, and/or WT1 were found negative, and BerEp4/CEA negative or focally positive.

### Whole-exome capture RNA sequencing

Exome-based RNA capture sequencing was carried out on FFPE samples for both rearranged and non-rearranged cases, as part of the MESOPATH/RENAPE diagnostic procedure, and retrieved retrospectively from our files. We targeted mesothelial proliferations with unusual clinical or pathological features. The data were analyzed to detect fusion genes and small nucleotide variations and to compare expression profiles with >6000 other samples using clustering methods. The molecular basis of the technique, the technical protocol, and the bioinformatic algorithms used have been described previously.^21^

In addition to the detection of gene fusions using the algorithms, the Arriba command-line tool was also used to detect other structural rearrangements with potential clinical relevance. Ward’s unsupervised clustering and Uniform Manifold Approximation and Projection (UMAP) were carried out in the R software (v4.2.2) environment, using the cluster and UMAP packages, respectively, on all cases of the cohort (*N* = 41, Supplementary Table S2, available at https://doi.org/10.1016/j.esmoop.2024.103644) for comparison with the transcriptomic data of other tumors retrieved retrospectively from our files, including angiomatoid fibrous histiocytoma (AFH, *N* = 18), solid and papillary mesothelial tumor (*N* = 12), pleural and peritoneal EM (*N* = 51, and *N* = 37, respectively), and SM (*N* = 40, and *N* = 2, respectively).

### Statistical analysis

The statistical analysis was carried out on the rearranged cases. Clinical and pathological data were compared using univariate analyses. Survival was investigated using the Kaplan–Meier method, log-rank test, and Cox regression analysis. The statistical software R with package RcmdrPlugin.EZR^22^ was used. A *P* value of <0.05 was considered significant.

## Results

### Clinical and histopathological characteristics of the cohort

**Figure 1:**
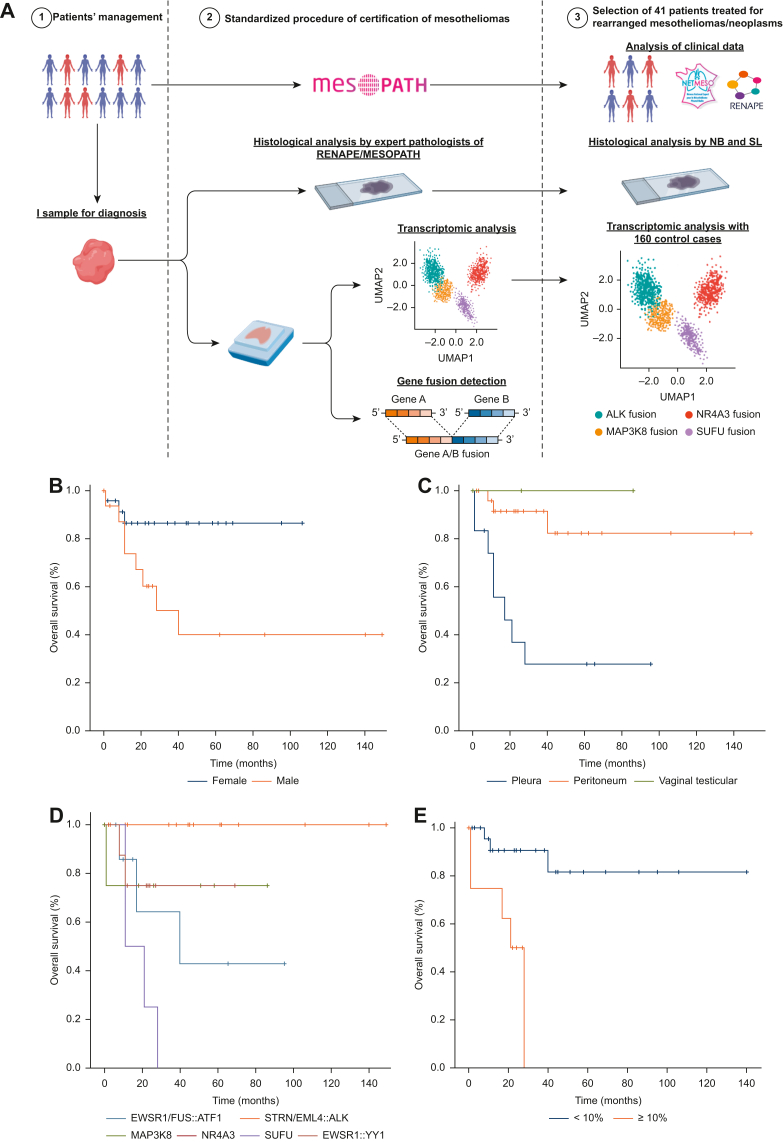
**Presentation of cohort and analysis of overall survival of cohort.** (A) Presentation of cohort. (B-E) Analysis of overall survival of cohort. The univariate analysis found that being female (*P* value: 0.0346, B), having a peritoneum or vaginal testicular (VT) site (*P* value: <0.001, C), harboring *ALK, MAP3K8*, or *NR4A3*-rearranged fusions (*P* value: 0.016, D), or exhibiting a Ki67 <10% (*P* value: <0.001, E) were associated with longer overall survival.

The 41 tumors with a fusion included 7 *ALK*-rearranged EM, 10 *MAP3K8*-rearranged EM, 4 *NR4A3*-rearranged EM, 8 epithelioid tumors harboring *EWSR1*::*FUS*/*ATF1 fusions*, 8 *EWSR1*::*YY1*-rearranged EM, and 4 *SUFU*-rearranged SM (Figure 1A). Their clinical features are presented in Table 1 (Figure 1A). The patients were treated based on the tumor site and therapeutic recommendations; for example, cytoreductive surgery plus hyperthermic intraperitoneal chemotherapy (CRS + HIPEC) for peritoneal epithelioid tumors or mesotheliomas. The median follow-up was 22 months (range: 1–149 months). The univariate analysis found that female sex (*P* value: 0.0346, Figure 1B), peritoneum or tunica vaginalis testis site (*P* value: <0.001, Figure 1C), *ALK*, or *NR4A3*-rearranged fusions (*P* value: 0.016, Figure 1D), or a Ki67 <10% (*P* value: <0.001, Figure 1E) were associated with a longer overall survival.

**Table 1:** Clinical characteristics and immunohistochemical features of the cohort (*N* = 41)

	*ALK* (*N* = 7)	*MAP3K8* (*N* = 10)	*EWSR1/FUS::ATF1* (*N* = 8)	*EWSR1*::*YY1* (*N* = 8)	*NR4A3* (*N* = 4)	*SUFU* (*N* = 4)
Clinical characteristics						
Sex (female), *N* (%)	7/7 (100.0)	6/10 (60.0)	5/8 (62.5)	5/8 (62.5)	1/4 (25.0)	1/4 (25.0)
Age (years), mean (range)	27.8 (11-37)	56.7 (30-89)	40.1 (6-87)	65.8 (51-84)	63.8 (60-68)	75.9 (66-84)
Presentation, *N* (%)						
Localized	0	1/10 (10.0)	2/8 (25.0)	4/8 (50.0)	1/4 (25.0)	1/4 (25.0)
Diffuse	7/7 (100.0)	9/10 (90.0)	6/8 (75.0)	4/8 (50.0)	3/4 (75.0)	3/4 (75.0)
Location, *N* (%)						
Peritoneum	7/7 (100.0)	5/10 (50.0)	5/8 (62.5)	7/8 (87.5)	4/4 (100.0)	0
Pleura	0	2/10 (20.0)	3/8 (37.5)	1/8 (12.5)	0	4/4 (100.0)
Tunica vaginalis testis	0	3/10 (30.0)	0	0	0	0
Treatment						
Peritoneal/vaginal site, *N* (%)	7/7 (100)	8/10 (80)	5/8 (62.5)	7/8 (87.5)	4/4 (100.0)	0
Neoadjuvant CT	5/7 (71.4)	1/8 (12.5)	2/5 (40.0)	0	0	
CRS ± HIPEC	6/7 (85.7)	4/8 (50.0)	4/5 (80.0)	6/7 (85.7)	2/4 (50.0)	
Adjuvant CT	2/7 (28.5)	0	2/5 (40.0)	1/7 (14.3)	1/4 (25.0)	
PCI, mean (range)	22.4 (15-28)	14.7 (5-20)	12.3 (3-27)	15.7 (10-26)	19.5 (13-26)	
CC score						
CC0-CC1	7/7 (100.0)	4/4 (100.0)	4/4 (100.0)	5/6 (83.3)	2/2 (100.0)	
CC2-CC3	0	0	0	1/6 (16.6)	0	
Pleural site, *N* (%)	0	2/10 (20)	3/8 (37.5)	1/8 (12.5)	0	4/4 (100.0)
Neoadjuvant CT		0	1/3 (33.3)	0		0
Adjuvant CT		2/2 (100.0)	3/3 (100.0)	1/1 (100.0)		4/4 (100.0)
PP ± HITeC		0	1/3 (33.3%)	0		0
Immunohistochemistry						
CKAE1-AE3	+++	+++	++/+++	+++^a^	+++	++/+++
Calretinin	++/+++	+++ (−/+)	- (++)	−/+ or +++	+++	+
CK5-6	+/++	- (+++)	Variable	- or +++	+/++	−/+
WT1	++/+++	+++ (−/+)	+++ (-)	- or +++	+++	−/+
BerEp4/CEA	-	-	- (+)	- (+)	-	-
PAX8	+++^a^	+++ (−/+)^a^	- (+)	-	-	−/++^b^
ERα	−/+	− (+)	0^c^	0^a^	-b	0^b^
p53	WT	WT	WT^c^	WT^c^	WT^c^	MT^c^
BAP1 (retained), *N* (%)	7/7 (100.0)	10/10 (100.0)	7/8 (87.5)	8/8 (100.0)	4/4 (100.0)	3/4 (75.0)
Ki67 (%) mean (range)	3.4 (3-5)	11.4 (1-40)	4.3 (1-20)^b^	4.2 (1-15)	2.3 (1-3)^a^	50.0 (40-60)^a^

CC score, completeness of cytoreduction score; CRS, cytoreductive surgery; CT, chemotherapy; HIPEC, hyperthermic intraperitoneal chemotherapy; HITeC, hyperthermic intra-thoracic chemotherapy; MT, mutant-type profile; PCI, peritoneal cancer index; PP, pleuropneumonectomy; WT, wild-type profile.

(), rarely observed; -, no expression of tumor cells; +, expression observed focally of tumor cells; ++, moderate expression in <50% of tumor cells; +++, high expression of tumor cells.

a Not available for one case.

b Not available for two cases.

c Not available for more than 2 cases.

#### *ALK*-rearranged EM (*N* = 7)

**Figure 2:**
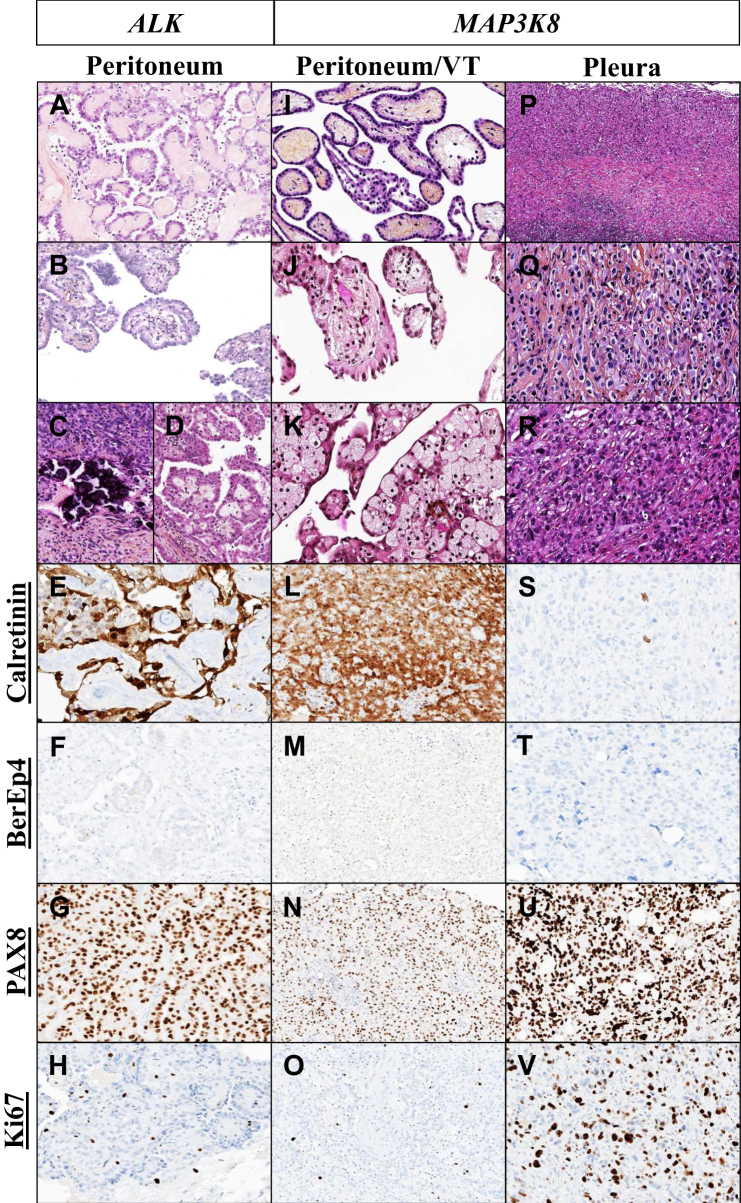
**Histological patterns and immunophenotype of mesothelioma rearranged- *ALK* or *MAP3K8*.***ALK*-rearranged cases (A-H) displayed extensive papillary proliferation of mesothelial cells, featuring hyaline or edematous papillae infiltrated by small-sized psammoma bodies and foamy cells. The mesothelial cells around the papillary axes appeared cuboidal or cylindrical with a consistent appearance (A-D). These cases exhibited classical mesothelial differentiation (E and F), conserving BAP1 and PAX8+ expression with a Ki67 proliferation index <5% (G-H). *MAP3K8*-rearranged peritoneal/vaginal testicular (VT) cases (I-O) resembled the *ALK* counterpart but showed a fringe-like cellular distribution around the papillae and a myxoid stroma within the papillae, distinguishing them (I-K). They also exhibited classical mesothelial differentiation (L and M), conserving BAP1 and PAX8+ expression, and a Ki67 proliferation index of <5% (N and O). *MAP3K8*-rearranged pleural cases (P-V) showed marked atypia, diffuse infiltration, and a doubtful mesothelial differentiation profile (S and T), yet maintained BAP1 and PAX8 expression with a Ki67 proliferation index of 40% (U and V).

This group was composed exclusively of females (7/7, 100%), and the mean age was significantly lower than in the other groups [27.8 years (range: 11-37 years), *P* value: <0.0001]. Tumors were multinodular and histologically they presented as extensive and diffuse papillary proliferations of mesothelial cells (Figure 2A) lining the peritoneal surface or superficially infiltrating the peritoneum. Notably, the mesothelial cells around the papillary axes, when clearly visible, appear cuboidal or cylindrical with a homogeneous appearance (Figure 2B). The papillae were edematous, focally infiltrated by foamy cells or psammoma (Figure 2C and D). Hyaline deposits were variably present within the stroma. Infiltration of the papillae was absent or superficial. Nuclear atypia was rare, there was approximately 1 mitosis per 2 mm^2^, and no necrosis. Tumor cells expressed a classical immunophenotype of mesothelial differentiation, with no BAP1 loss. Nuclear PAX8 staining was always present and Ki67 was <5% (Figure 2E-H). Four were treated with a combination of cisplatin or carboplatin and pemetrexed, and one by an anaplastic lymphoma kinase (ALK) inhibitor (alectinib) before surgery. Five patients were treated by CRS + HIPEC. Two of them were treated with carboplatin and paclitaxel or with a programmed cell death protein 1 (PD-L1) inhibitor (durvalumab) as adjuvant therapy. Among those without HIPEC, one underwent neoadjuvant chemotherapy with a combination of cisplatin and pemetrexed, followed by CRS only. The last patient underwent regular surveillance without surgical or chemotherapeutical intervention (Table 1). This group had a prolonged overall survival; there was no death nor recurrence over a mean follow-up of 56.7 months (range: 8-149 months).

#### *MAP3K8*-rearranged EM (*N* = 10)

These patients were predominantly female (6/10, 60%), and the mean age was 56.7 years (range: 30-89 years). Half (5/10, 50%) of the cases arose from the peritoneum, 30% (3/10) from the tunica vaginalis testis, and 20% (2/10) from the pleura. The disease presentation was characterized by a multinodular spread, except for one case that was localized. Two histological patterns were observed according to the site of the proliferations. In the peritoneum/tunica vaginalis testis, tumors closely resembled its *ALK*-rearranged counterpart, displaying extensive and diffuse papillary proliferation of mesothelial cells (Figure 2I). A distinguishing feature was the fringe-like cellular distribution around the papillae (Figure 2J), setting it apart from the typical pattern observed in *ALK* fusion cases. The papillae contained a myxoid stroma associated with sometimes foamy cells and/or psammoma focally (Figure 2K) as well. Variable hyaline deposits were present within the stroma. Infiltrative propensity was either absent or superficial. All tumors were low grade, without necrosis or severe atypia, and there was approximately 1 mitosis per 2 mm^2^. IHC revealed a classical profile of mesothelial differentiation with no loss of BAP1. PAX8 was almost consistently expressed (6/7, 85.7%), with a Ki67 proliferation index of <5% (Table 1, Figure 2L-O). The two pleural cases appeared more aggressive, with marked atypia and a diffuse sheet-like fashion infiltration of the pleural tissue (Figure 2P-R), which was sometimes associated with a dense chronic inflammation at the periphery of the tumor. The nuclear grade was severe and the mean mitotic count was 5 per 2 mm^2^. No foamy cells, psammoma bodies, or necrosis were recognized. The tumors had a doubtful profile of mesothelial differentiation and no BAP1 loss. PAX8 was consistently expressed (2/2, 100%), with a Ki67 proliferation index of 40% (Table 1, Figure 2S-V). For peritoneal cases, the patients were treated by a combination of cisplatin and pemetrexed followed by a CRS for one case (1/5, 20%), by a CRS for one case (1/5, 20%), and with CRS + HIPEC for two cases (2/5, 40%); one was not treated due to his age (1/5, 20%). The cases of tunica vaginalis testis were treated by surgical resection (3/3, 100%; Table 1). This group of peritoneal and vaginal sites had an intermediate overall survival; there was no death over a mean follow-up of 38 months (range: 3-86 months) and one recurrence at 58 months. In contrast, the two pleural cases had an extremely unfavorable outcome, patients succumbing to their illness in the month following diagnosis.

#### *EWSR1/FUS::ATF1*-rearranged epithelioid tumors (*N* = 8)

**Figure 3:**
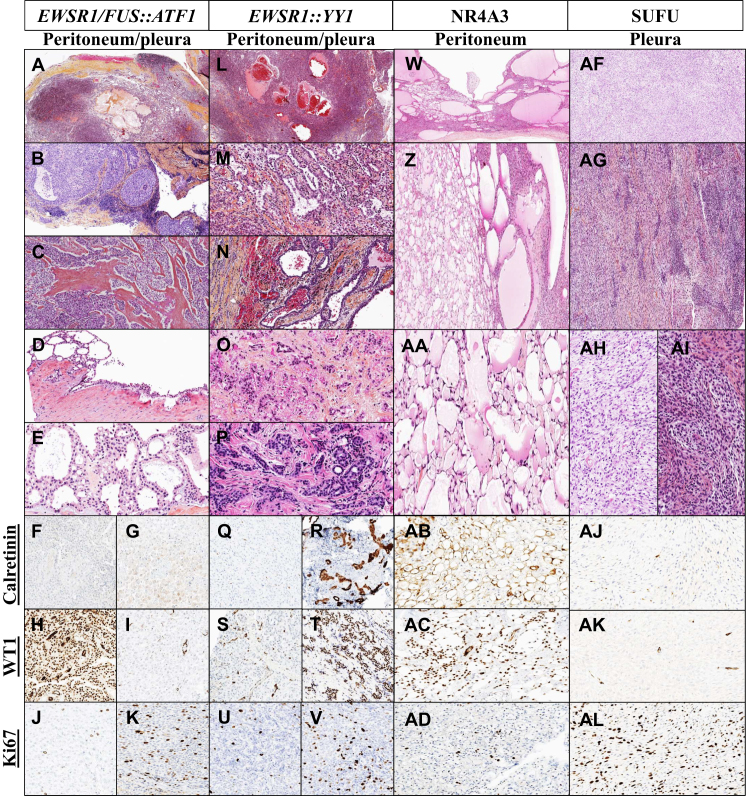
**Histological patterns and immunophenotype of neoplasms rearranged-*ATF1*, *ESWSR1, NR4A3*, and *SUFU*.***EWSR1/FUS*::*ATF1*-rearranged cases (A-K) displayed well-circumscribed neoplasms within a fibrous capsule and dense lymphoid cuffing (A and B). The predominant solid architectural pattern was observable alongside macrocysts and microcysts containing serous fluid (C-E). Mesothelial differentiation was doubtful (F-I). BAP1 was retained, PAX8 was negative, and the Ki67 proliferation index ranged from <5% to 10%-20% (J and K). *ESWSR1*::*YY1*-rearranged cases (L-V) displayed scattered tumor nodules and epithelioid cells arranged in cords and trabeculae (L-P). Some cases resembled angiomatoid fibrous histiocytoma-like tumors (L). Immunohistochemically, 75% exhibited classical mesothelial profile, while 25% had a doubtful profile (Q-T). BAP1 was retained, PAX8 was negative, and Ki67 proliferation ranged between <10% and 15%-30% (U and V). *NR4A3*-rearranged cases (W-AD) displayed a constant microcystic architecture (W-AA) without atypia, mitotic activity, or necrosis. The transition into spindle cell proliferation occupied <10% of the tumor surface (W). These cases showed a classical mesothelial differentiation profile (AB and AC). BAP1 was retained, PAX8 was negative, and the Ki67 proliferation index was <5% (AD). *SUFU*-rearranged cases (AF-AL) exhibited a uniform appearance with hypo- and hypercellular areas (AF-AG), occasionally displaying a chevron-like pattern. Spindle-shaped cells arranged in a bland fashion within a fibrous or myxoid stroma were moderately atypical (AH and AI). BAP1 was retained, PAX8 was positive in one case, and the Ki67 index ranged from 40% to 60% (AJ-AL).

These patients were predominantly female (5/8, 62.5%), and the mean age was 40 years (range: 6-87 years). More than half of the cases arose from the peritoneum (5/8, 62.5%), and the others from the pleura. A multinodular spread was observed in 6/8 cases (75%). In six cases, well-circumscribed neoplasms enclosed within a thick fibrous capsule, often accompanied by dense lymphoid cuffing were observed (Figure 3A and B). The predominant architectural pattern was solid (Figure 3C), although macrocysts and microcysts (Figure 3D) were discernible, with the smaller cysts containing serous fluid (Figure 3E). In two cases, the tumor was composed of epithelioid cells arranged in a sheet-like pattern or forming small tubular structures surrounding serous fluid. At higher magnification, the epithelioid cells exhibited ill-defined borders, eosinophilic cytoplasm, relatively round but slightly irregular nuclear membranes, vesicular chromatin with prominent nucleoli, and rare mitotic figures. The tumors had a doubtful profile of mesothelial differentiation, with a low or absent calretinin expression. BAP1 loss was observed in 1 case (1/8, 12.5%). PAX8 expression was focal in only 1 case (1/8, 12.5%). The Ki67 proliferation index was <5% in 5/6 cases, and in 1 case this ranged from 10% to 20% (Figure 3F-K). For peritoneal cases, 3 patients were treated by CRS + HIPEC (3/5, 60%), and 1 of them received a combination of cisplatin and pemetrexed as adjuvant treatment (1/5, 20%). The remaining 2 patients were treated by CRS only without any adjuvant treatment (2/5, 40%; Table 1). This group displayed intermediate overall survival; there were two deaths and two instances of recurrence recorded. The mean follow-up was 16.2 months (range: 8-40 months). For pleural cases, only one patient succumbed to their illness 18 months after diagnosis, and two recurrences were reported at 10 and 65.3 months after diagnosis. Two cases were treated with conventional chemotherapy, with or without external radiotherapy, while the third was treated with tocilizumab followed by pleuropneumonectomy and HIPEC, and after surgery, continued with tocilizumab. Conversely, pleural cases exhibited better overall survival; the mean follow-up was 32.1 months (range: 13-65.3 months).

#### *EWSR1::YY1*-rearranged EM (*N* = 8)

Patients were predominantly female (5/8, 62.5%), and the mean age was 65.8 years (range: 51-84 years). Tumors were found predominantly in the peritoneum (7/8, 87.5%). The disease presentation was characterized in half of the cases by a multinodular spread (4/8, 50%). Microscopic examination revealed, at low magnification, small tumor nodules scattered throughout, sometimes accompanied by prominent lymphoid aggregates (Figure 3L). The predominant histological pattern consisted of epithelioid cells arranged in cords and trabeculae, some with a macrocystic or focally papillary appearance, set against a background of abundant stromal collagen or lightly myxoid stroma (Figure 3M-P). Additionally, three cases exhibited areas with distinct cell arrangements and extensive regions with solid nests and sheets, occasionally displaying pseudocystic structures containing hemorrhagic material or eosinophilic fluid, reminiscent of AFH-like tumors (Figure 3L). Cytologically, tumor cells were large, polygonal, monomorphic epithelioid cells with well-defined cell borders, centrally located nuclei, with a fine chromatin, and inconspicuous nucleoli. Nuclear-to-cytoplasmic ratios varied among the cases. Neither mitotic activity nor tumor necrosis was observed. Immunohistochemical examination generally revealed a classical profile of mesothelial differentiation (6/8, 75%) and a doubtful profile in 25% of cases (2/8, 25%). BAP1 expression was consistently preserved (7/7, 100%) and PAX8 expression was negative (8/8, 100%). The Ki67 was <5% in 5/8 cases; in 3 cases this ranged from 5% to 15% (Figure 3Q-V). All but 1 patient with peritoneal disease were treated by CRS alone or in combination with HIPEC (6/7, 85.7%); the remaining patient was an elderly patient for whom surgery was not considered (1/7, 14.3%). Adjuvant treatment with carboplatin and pemetrexed was proposed for 1 patient (1/7, 14.3%; Table 1). This group exhibited intermediate overall survival; one patient died 11 months after diagnosis despite receiving only CRS, and no recurrence was observed during a mean follow-up of 26.8 months (range: 11-69 months). Conversely, in the pleural case, the patient experienced extremely poor survival outcome, succumbing to his illness 8 months after diagnosis.

#### *NR4A3*-rearranged EM (*N* = 4)

These patients were predominantly male (3/4, 75%), and the mean age was 63.8 years (range: 60-68 years). All tumors arose from the peritoneum and were predominantly multinodular and exophytic. Histologically, microcystic architecture was constant, with cysts lined by a single layer of flattened mesothelial cells arranged diffusely in a sieve-like configuration, with limited stroma and eosinophilic secretion (Figure 3W-AA). Notably, there was no cytological atypia, mitotic activity, or necrosis. Additionally, a sharp transition was observed as the microcystic pattern transformed into spindle cell proliferation (within <10% of the tumor surface, Figure 3W). The tumors had a classical profile of mesothelial differentiation. It is worth noting that BAP1 expression remained intact in all cases (4/4, 100%), with no PAX8 expression (4/4, 100%) and low Ki67 <5% (Table 1, Figure 3AB-AD). All patients underwent surgical resection; 2 of them received HIPEC (2/4, 50%), among whom 1 also received a combination of cisplatin and doxorubicin due to a high peritoneal cancer index (peritoneal cancer index = 26, 1/4, 25%). Among the other 2 patients, HIPEC or chemotherapy were not administered due to either treatment refusal or management in a non-expert peritoneal mesothelioma center (2/4, 50%). This group exhibited a prolonged overall survival with no death or recurrence, and the mean follow-up was 60.8 months (range: 14-140 months). Among them, the two patients without adjuvant treatment had a mean follow-up of 101 months (range: 62-140 months).

#### *SUFU*-rearranged SM (*N* = 4)

**Figure 4:**
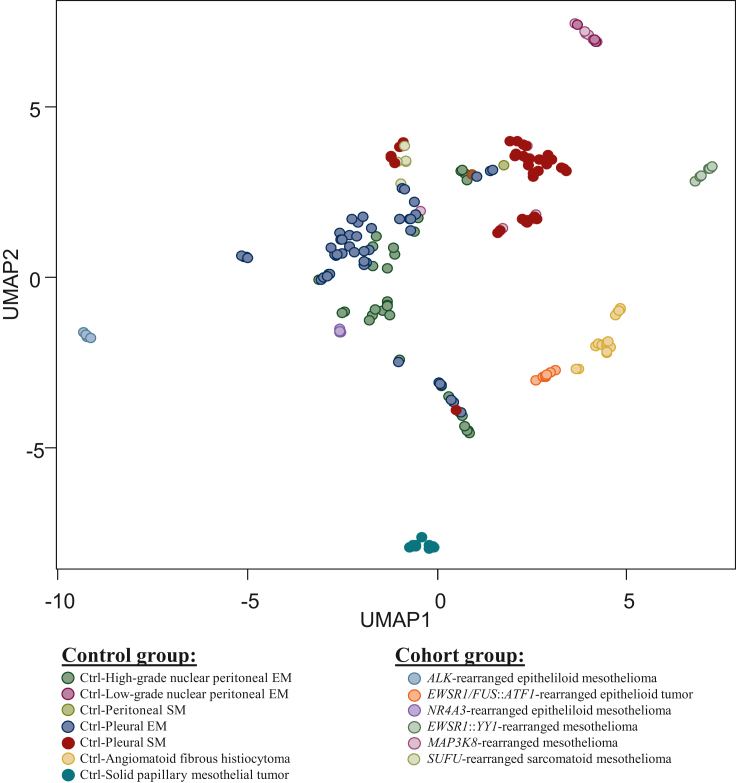
**Analysis of UMAP projection.** The clustering analysis using gene expression profiling grouped: cases with gene fusions (*N* = 41) versus control cases without gene fusions (*N* = 160). The distinct subgroups emerged: *ALK*, *NR4A3*, or *MAP3K8*-rearranged mesotheliomas formed separate clusters based on histology and site. *EWSR1/FUS*::*ATF1*-rearranged epithelioid tumors constituted a distinct group from angiomatoid fibrous histiocytoma, while *EWSR1*::*YY1*-rearranged mesotheliomas formed their unique cluster. *SUFU*-rearranged sarcomatoid mesothelioma clustered with some pleural mesotheliomas, distinctly different from sarcomas and carcinomas in the database. Papillary and solid mesothelial tumors exhibited a unique transcriptomic profile compared with other rearranged proliferations. Ctrl, control; EM, epithelioid mesothelioma; SM, sarcomatoid mesothelioma; UMAP, Uniform Manifold Approximation and Projection.

These patients were predominantly male (3/4, 75%), and the mean age was 75.9 years (range: 66-84 years). Tumors exclusively arose from the pleura (4/4, 100%). Some 3 of the 4 cases had a multinodular spread (3/4, 75%); 1 had an isolated lesion (1/4, 25%). All cases demonstrated a remarkably uniform appearance at low magnification, presenting as a cell culture-like proliferation with alternating hypo- and hypercellular areas (Figure 4AF and AG). Some cases exhibited a chevron-like pattern. At higher magnification, spindle-shaped cells were moderately atypical, arranged in a bland fashion within a fibrous or myxoid stroma (Figure 4AH and AI). Their cytoplasmic border was scarcely visible; their nucleus displayed a fine chromatin and some barely visible nucleoli. Neither necrosis nor calcification was observed. Their immunohistochemical profile was not strongly indicative of a mesothelial lineage. Additionally, BAP1 expression was lost in 3 cases (3/4, 75%). PAX8 was assessed in 1 case and found to be positive (1/4, 25%), and the Ki67 index ranged from 40% to 60% (Table 1, Figure 4AJ-AL). All patients were treated with a combination of chemotherapy (4/4, 100%; pemetrexed with vinorelbine or bevacizumab/carboplatin; Table 1). Unfortunately, within this group, all patients eventually succumbed to their illness without observed prolonged stabilization of their lesions; the mean follow-up was 17.5 months (range: 11-28 months).

### Clustering analyses

Clustering analysis using gene expression profiling included cases with gene fusions and control cases without gene fusions. The UMAP projection showed that all *ALK*, *NR4A3*, and *MAP3K8*-rearranged EM formed distinct subgroups, with transcriptomic similarities based on the histology and the site of mesothelioma. *EWSR1*/*FUS*::*ATF1*-rearranged epithelioid tumors clustered together, constituting a separate group from AFH. Similarly, *EWSR1*::*YY1*-rearranged EM all clustered together and stood out from the other neoplasms. *SUFU*-rearranged SM clustered with some epithelioid/sarcomatoid pleural mesotheliomas (Figure 4). They were also distinct from the sarcomas and carcinomas of the Leon Berard Cancer Center transcriptomic database (data not shown) providing strong evidence of their mesothelial lineage. No mutation was identified, except for two pleural *MAP3K8*-rearranged cases with *TP53* mutation, as well as three *SUFU*-rearranged cases with either *BAP1* mutation or *PI3KCA*/*BAP1* co-mutation. Additionally, the analysis of papillary and solid mesothelial tumors harbored a divergent transcriptomic profile.

## Discussion

We have expanded herein the spectrum of rearranged mesothelial tumors and defined novel epithelioid (mesothelial) proliferations with distinct histological growth patterns associated with oncological courses. We found a female predominance, except for cases harboring *NR4A3* and *SUFU* malignant mesothelioma, and most patients were around 60 years at diagnosis, but those harboring *ALK* or *EWSR1/FUS::ATF1* gene fusions were younger.

When located in the peritoneum, *ALK* and *MAP3K8*-rearranged EM were characterized by a typical papillary growth pattern, often superficial. They were frequently associated with foamy cells and/or psammoma in peritoneal and vaginal testicular sites, with *MAP3K8*-rearranged mesothelioma often displaying fringe-like cellular distribution of the cylindrical tumor cells around the papillae, as seen in a case report.^13^ They all expressed a classical profile of mesothelial differentiation and a PAX8 expression. They presented as low-grade malignancies and patients had a very long overall survival. In contrast, *MAP3K8-*rearranged pleural proliferations harboring *TP53* mutation appeared as poorly differentiated tumors. The overall survival was pejorative; patients succumbed to their illness within 1 month of diagnosis.

*NR4A3*-rearranged tumors have been recently described in a series of five cases and well characterized histologically.^18^ This subset of tumors, both herein and in the recently published paper, presents as a unique microcytic lesion closely resembling adenomatoid tumors, but there was no mutation of *TRAF7* or *CDC42* which is characteristic of the latter.^23^

*EWSR1*::*YY1* and *EWSR1*/*FUS*::*ATF1-*rearranged lesions represent emerging entities, and the debate about their nature is still ongoing.^7^^,^^9^, ^10^, ^11^, ^12^^,^^14^, ^15^, ^16^^,^^24^ The *EWSR1*::*YY1* and *EWSR1****/****FUS*::*ATF1-*rearranged tumors reported herein typically presented as cystic nodules that have AFH-like features. Although *EWSR1*::*ATF1* and *FUS*::*ATF1* fusions are reported in clear-cell sarcomas and AFH,^25^^,^^26^ the cases herein with *EWSR1*/*FUS*::*ATF1* fusions were very close to the AFH cluster, but clearly separated on the UMAP projection. The other mesotheliomas and *EWSR1*::*YY1*-rearranged lesions were more distant from the AFH cluster. This disagrees with what was reported by the Antonescu group who found a loose clustering of conventional mesotheliomas, *EWSR1*::*YY1* fused lesions (*N* = 2), and *EWSR1*/*FUS*::*ATF1* fused lesions (*N* = 3).^12^ Their study was based on their methylation profiling and included a large number of mesotheliomas (*N* = 79) retrieved from the Heidelberg mesothelioma study (GSE164269), and from The Cancer Genome Atlas (50 colorectal adenocarcinoma, 50 lung adenocarcinoma, and 73 mesotheliomas). Their analysis was discriminant enough to separate those mesothelial lesions from the other malignant tumors, but not sufficient to decipher the spectrum of those rare rearranged mesothelial tumors.^12^ We demonstrated herein that *EWSR1*/*FUS*::*ATF1* fused tumors should probably be considered as distinct mesothelial tumors. This is crucial because these tumors often exhibit an incomplete mesothelial immunophenotype, with no or little calretinin, and variable CK5-6 and WT1 expressions, as previously reported.^7^, ^8^, ^9^, ^10^^,^^12^^,^^14^^,^^15^^,^^24^

Lastly, for the group harboring *SUFU* gene fusion, the histological appearance is deceptively reassuring, with monotonous spindle-shaped cells within a homogeneous fibrous or myxoid stroma. As the immunohistochemical profile was inconclusive for mesothelial proliferation, except for BAP1 loss, the diagnosis of mesothelioma was established mainly based on their transcriptomic profile. Interestingly these tumors form a distinct cluster with pleural sarcomatoid mesotheliomas and exhibit a short overall survival. Of note, SUFU as GLI1-3 and SMO are involved in the hedgehog signaling pathway which plays a crucial role in regulating normal cell growth and differentiation. When this pathway is not properly controlled, however, it can lead to abnormal hedgehog signaling, resulting in the development and progression of various aggressive human cancers. This includes neoplastic transformation, tumor progression, metastasis, and drug resistance. Abnormal activation of the hedgehog pathway has been associated with various cancers. As SUFU negatively regulates the hedgehog pathway,^27^ targeting the abnormal activity of SUFU holds promise as a therapeutic strategy to impact GLI1, a pivotal contributor to the advancement of multiple cancer types via the hedgehog signaling pathway. It is important to note, however, that as of now, this approach has shown promise in cellular and animal models^28^, ^29^, ^30^ and a few cases of clinically established mesotheliomas, due to the low frequency of the hedgehog signaling pathway alterations.^31^

### Conclusion

This study significantly broadens the spectrum of mesothelial tumors associated with fusions, offering insight into novel epithelioid (mesothelial) proliferations with distinctive histological appearances, molecular profiles, and prognoses to guide adapted treatments for patients.

## References

[1] Robinson B.W.S., Lake R.A. (2005). Advances in malignant mesothelioma. N Engl J Med.

[2] Tsao A.S., Lindwasser O.W., Adjei A.A. (2018). Current and future management of malignant mesothelioma: a consensus report from the National Cancer Institute Thoracic Malignancy Steering Committee, International Association for the Study of Lung Cancer, and Mesothelioma Applied Research Foundation. J Thorac Oncol.

[3] Opitz I., Bille A., Dafni U. (2023). European epidemiology of pleural mesothelioma—real-life data from a Joint Analysis of the Mesoscape Database of the European Thoracic Oncology Platform and the European Society of Thoracic Surgery Mesothelioma Database. J Thorac Oncol.

[4] Hmeljak J., Sanchez-Vega F., Hoadley K.A. (2018). Integrative molecular characterization of malignant pleural mesothelioma. Cancer Discov.

[5] Bueno R., Stawiski E.W., Goldstein L.D. (2016). Comprehensive genomic analysis of malignant pleural mesothelioma identifies recurrent mutations, gene fusions and splicing alterations. Nat Genet.

[6] Zauderer M.G., Martin A., Egger J. (2021). The use of a next-generation sequencing-derived machine-learning risk-prediction model (OncoCast-MPM) for malignant pleural mesothelioma: a retrospective study. Lancet Digit Health.

[7] Desmeules P., Joubert P., Zhang L. (2017). A subset of malignant mesotheliomas in young adults are associated with recurrent EWSR1/FUS-ATF1 fusions. Am J Surg Pathol.

[8] Argani P., Harvey I., Nielsen G.P. (2020). EWSR1/FUS–CREB fusions define a distinctive malignant epithelioid neoplasm with predilection for mesothelial-lined cavities. Mod Pathol.

[9] Dermawan J.K., Vanoli F., Herviou L. (2022). Comprehensive genomic profiling of EWSR1/FUS::CREB translocation-associated tumors uncovers prognostically significant recurrent genetic alterations and methylation-transcriptional correlates. Mod Pathol.

[10] Trecourt A., Macagno N., Ngo C. (2023). CREB fusion–associated epithelioid mesenchymal neoplasms of the female adnexa: three cases documenting a novel location of an emerging entity and further highlighting an ambiguous misleading immunophenotype. Virchows Arch.

[11] Shibayama T., Shimoi T., Mori T. (2022). Cytokeratin-positive malignant tumor in the abdomen with EWSR1/FUS-CREB fusion: a clinicopathologic study of 8 cases. Am J Surg Pathol.

[12] Dermawan J.K., Torrence D., Lee C. (2022). *EWSR1 :: YY1* fusion positive peritoneal epithelioid mesothelioma harbors mesothelioma epigenetic signature: report of 3 cases in support of an emerging entity. Genes Chromosomes Cancer.

[13] Panagopoulos I., Andersen K., Brunetti M. (2023). Genetic pathways in peritoneal mesothelioma tumorigenesis. Cancer Genomics Proteomics.

[14] Ren H., Rassekh S.R., Lacson A. (2021). Malignant mesothelioma with *EWSR1-ATF1* fusion in two adolescent male patients. Pediatr Dev Pathol.

[15] Ke H., Gill A.J., McKenzie C. (2021). Malignant peritoneal mesothelioma with EWSR1-ATF1 fusion: a case report. JTO Clin Res Rep.

[16] Kimpo M.S., Francisco K.L., Chong Q.T. (2022). Mesothelioma with ALK gene mutations in two pediatric patients: clinical course and outcome. Pediatr Blood Cancer.

[17] Javaid S., Patton A., Tinoco G., Oghumu S., Iwenofu O.H. (2023). Metastatic sporadic paraganglioma with *EWSR1::CREM* gene fusion: a unique molecular profile that expands the phenotypic diversity of the molecular landscape of the *EWSR1::CREM* gene fusion positive tumors. Genes Chromosomes Cancer.

[18] Agaimy A., Brcic L., Briski L.M. (2023). NR4A3 fusions characterize a distinctive peritoneal mesothelial neoplasm of uncertain biological potential with pure adenomatoid/microcystic morphology. Genes Chromosomes Cancer.

[19] Argani P., Lian D.W.Q., Agaimy A. (2021). Pediatric mesothelioma with ALK fusions: a molecular and pathologic study of 5 cases. Am J Surg Pathol.

[20] (2021). WHO Classification of Tumours Editorial Board.

[21] Macagno N., Pissaloux D., De La Fouchardière A. (2022). Wholistic approach: transcriptomic analysis and beyond using archival material for molecular diagnosis. Genes Chromosomes Cancer.

[22] Kanda Y. (2013). Investigation of the freely available easy-to-use software ‘EZR’ for medical statistics. Bone Marrow Transplant.

[23] Goode B., Joseph N.M., Stevers M. (2018). Adenomatoid tumors of the male and female genital tract are defined by TRAF7 mutations that drive aberrant NF-kB pathway activation. Mod Pathol.

[24] Panagopoulos I., Thorsen J., Gorunova L. (2013). RNA sequencing identifies fusion of the *EWSR1* and *YY1* genes in mesothelioma with t(14;22)(q32;q12). Genes Chromosomes Cancer.

[25] Mae H., Outani H., Imura Y. (2023). Targeting the clear cell sarcoma oncogenic driver fusion gene *EWSR1::ATF1* by HDAC inhibition. Cancer Res Commun.

[26] Antonescu C.R., Dal Cin P., Nafa K. (2007). EWSR1-CREB1 is the predominant gene fusion in angiomatoid fibrous histiocytoma. Genes Chromosomes Cancer.

[27] Kato S., Tomson B.N., Buys T.P.H., Elkin S.K., Carter J.L., Kurzrock R. (2016). Genomic landscape of malignant mesotheliomas. Mol Cancer Ther.

[28] Meerang M., Bérard K., Felley-Bosco E. (2016). Antagonizing the hedgehog pathway with vismodegib impairs malignant pleural mesothelioma growth in vivo by affecting stroma. Mol Cancer Ther.

[29] Doheny D., Manore S.G., Wong G.L., Lo H.W. (2020). Hedgehog signaling and truncated GLI1 in cancer. Cells.

[30] You M., Varona-Santos J., Singh S., Robbins D.J., Savaraj N., Nguyen D.M. (2014). Targeting of the Hedgehog signal transduction pathway suppresses survival of malignant pleural mesothelioma cells in vitro. J Thorac Cardiovasc Surg.

[31] Popat S., Sharma B., MacMahon S. (2021). Durable response to vismodegib in PTCH1 F1147fs mutant relapsed malignant pleural mesothelioma: implications for mesothelioma drug treatment. JCO Precis Oncol.

