# Supplementary material for: Update on gene fusions and the emerging clinicopathological landscape of peritoneal and pleural mesotheliomas and other neoplasms
Source: ESMO Open. 2024 Jul 25;9(8):103644. doi: 10.1016/j.esmoop.2024.103644 (PMC11326890; doi:10.1016/j.esmoop.2024.103644)
Supplement: Supplementary data [file mmc1.docx]

**SUPPLEMENTARY TABLES:**

**Supplementary Table S1: Details of the antibodies used for immunohistochemistry.**

| Antibody | Clone | Dilution | Manufacturer |
| --- | --- | --- | --- |
| CEA | II-7 | 1:100 | Dako |
| BAP1 | C-4 | 1:100 | Histopathology |
| BerEP4 | nazi | 1:200 | Dako |
| Calretinin | Polyclonal | 1:50 | Zymed |
| ERα | EP1 | 1:1 | Dako |
| CK5-6 | D5/16B4 | 1:50 | Dako |
| CK7 | OV-TL 12/30 | 1:100 | Dako |
| CKAE1/AE3 | AE1AE3 | 1:50 | Dako |
| Ki67 | SP6 | 1:100 | NeoMarkers |
| PAX8 | Polyclonal | 1:25 | Zytomed Systems |
| WT1 | 6F-H2 | 1:1 | Dako |

ERα, Estrogen Receptor α

**Supplementary Table S2: Details of molecular alterations.**

| ID | Gene fusion | Mutations |
| --- | --- | --- |
| 1 | *STRN*::*ALK* |  |
| 2 | *STRN*::*ALK* |  |
| 3 | *STRN*::*ALK* |  |
| 4 | *STRN*::*ALK* |  |
| 5 | *EML4*::*ALK* |  |
| 6 | *STRN*::*ALK* |  |
| 7 | *EML4*::*ALK* |  |
| 8 | *FUS*::*ATF1* |  |
| 9 | *EWSR1*::*ATF1* |  |
| 10 | *EWSR1*::*ATF1* |  |
| 11 | *EWSR1*::*ATF1* |  |
| 12 | *EWSR1*::*ATF1* |  |
| 13 | *EWSR1*::*ATF1* |  |
| 14 | *EWSR1*::*ATF1* |  |
| 15 | *EWSR1*::*ATF1* |  |
| 16 | *EWSR1*::*YY1* |  |
| 17 | *EWSR1*::*YY1* |  |
| 18 | *EWSR1*::*YY1* |  |
| 19 | *EWSR1*::*YY1* |  |
| 20 | *EWSR1*::*YY1* |  |
| 21 | *EWSR1*::*YY1* |  |
| 22 | *EWSR1*::*YY1* |  |
| 23 | *EWSR1*::*YY1* |  |
| 24 | *MAP3K8*::*AFAP1L2* |  |
| 25 | *MAP3K8*::*ANKRD22*, *MAP3K8*::*TFDP2* |  |
| 26 | *MAP3K8*::*AFAP1L2* |  |
| 27 | *MAP3K8*::*SVIL* |  |
| 28 | *MAP3K8.ARHGEF18* |  |
| 29 | *MAP3K8*::*FAM117A* |  |
| 30 | *MAP3K8*::*SPAG16* |  |
| 31 | *MAP3K8*::*VRK2* | *TP53* |
| 32 | *MAP3K8*::*PITRM1* |  |
| 33 | *MAP3K8*::*MTPAP* | *TP53* |
| 34 | *EWSR1*::*NR4A3* |  |
| 35 | *EP300*::*NR4A3* |  |
| 36 | *EP300*::*NR4A3* |  |
| 37 | *SEC24B*::*NR4A3* |  |
| 38 | *SUFU*::*ADK* | *BAP1*, *PI3K3CA* |
| 39 | *SUFU*::*LINC01164* | *BAP1* |
| 40 | *SUFU*::*LINC01164* | *BAP1* |
| 41 | *SUFU*::*WBP1L* |  |
